# Case report: Unusual coexistence between familial hypercholesterolemia and familial hypobetalipoproteinemia

**DOI:** 10.3389/fcvm.2022.942772

**Published:** 2022-08-08

**Authors:** Kei Sasaki, Hayato Tada, Masa-aki Kawashiri, Toshimitsu Ito

**Affiliations:** ^1^Department of Internal Medicine, Self-Defense Forces Central Hospital, Tokyo, Japan; ^2^Division of Anti-aging, Department of Internal Medicine, National Defense Medical College, Tokorozawa, Japan; ^3^Division of Cardiovascular Medicine, Kanazawa University Graduate School of Medicine, Kanazawa, Japan

**Keywords:** familial hypercholesterolemia, familial hypobetalipoproteinemia, LDL cholesterol, apolipoprotein B, PCSK9

## Abstract

Type 1 familial hypobetalipoproteinemia (FHBL1), characterized by low levels of apolipoprotein B (ApoB)-containing lipoproteins, elevation of transaminases, and hepatic steatosis, is a rare disease the prevalence of which is 1 in 3,000 among general population. Here we report an extremely rare family where phenotypes of familial hypercholesterolemia (FH) are canceled by coexistence of FHBL1 caused by an truncating mutation in apolipoprotein B (*APOB*).

## Introduction

Type 1 familial hypobetalipoproteinemia (FHBL1, OMIM #615558) is a rare disorder exhibiting very low LDL cholesterol due to genetic mutations in apolipoprotein B (*APOB*) (1, 2). One of the main manifestations of this disease includes hepatic dysfunction (3). It is usually difficult to diagnose this disease correctly, since variety of other situations and/or diseases, such as hyperthyroidism, malnutrition, liver dysfunction, and lipid lowering therapies could mimic it. On the other hand, familial hypercholesterolemia (FH) is also a genetic condition where disturbance of LDL metabolism leads to elevation of LDL cholesterol and premature coronary artery disease (4, 5). The prevalence of FHBL1 is estimated to be around 1 in 3,000 among general population (6), and that of FH is 1 in 300 among general population (7). These are different genetic diseases whose effects on LDL cholesterol and coronary artery disease are opposite (8). We encountered a rare family where a rare coincidence of FHBL1 and FH coexists. In fact, comprehensive genetic analysis using a custom lipid panel of 21 genes associated with Mendelian lipid disorders was quite useful for us to uncover the complicated situations in this family.

## Case descriptions

### Lipid measurements

Serum levels of total cholesterol, triglycerides, and HDL cholesterol were determined enzymatically using automated instrumentation. LDL cholesterol level was calculated using the Friedewald formula.

### Genetic analysis

We sequenced the coding regions of 21 genes known to be associated Mendelian lipid disorders using the custom panel as previously described on six family members (9). A rare variant (the frequency of which is < 1%) was considered as pathogenic mutation if it fulfilled any of the following criteria: (1) protein truncated mutations, or (2) registered as pathogenic in Clinvar.

### Ethical considerations

The study was approved by the Ethics Committee at the Kanazawa University and National Cerebral and Cardiovascular Center Research Institute. All procedures followed were in accordance with the ethical standards of the responsible committee on human experimentation (institutional and national) and with the Helsinki Declaration of 1975, as revised in 2008. Informed consent for genetic analyses was obtained from all the subjects for inclusion in the study.

### Case descriptions

The proband was a 50-year-old male who had a history of Graves' disease at the age of 40. During his clinical course, his disease had also been complicated with hypobetalipoproteinemia, with elevated transaminases (aspartate aminotransferase = 38 IU/L, alanine aminotransferase = 51 IU/L) and quite low LDL cholesterol levels (19 mg/dL). He was also diagnosed with non-alcoholic fatty liver disease based on findings of a severe bright liver on abdominal ultrasound.

**Figure 1:**
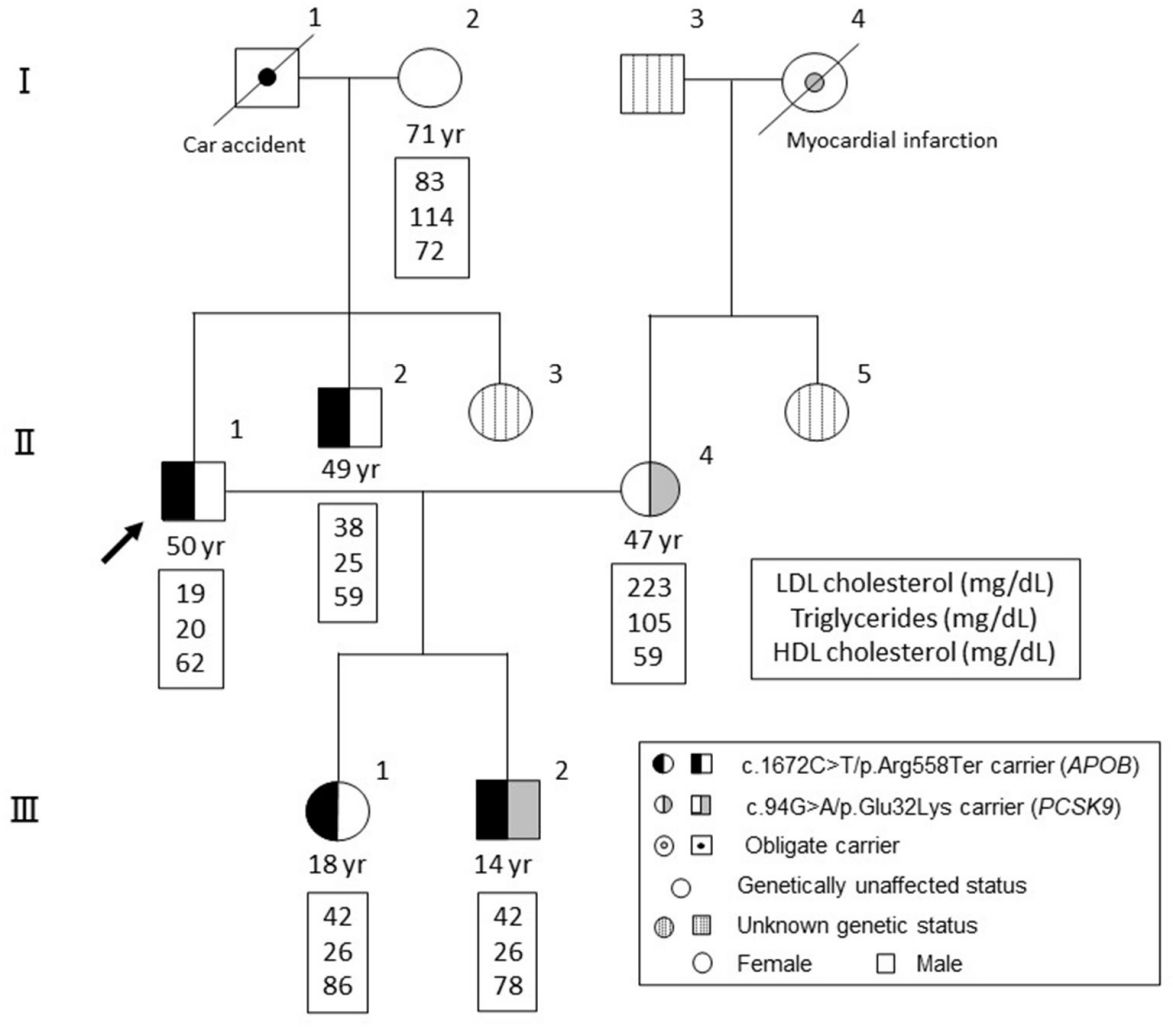
Family tree. Black denotes individuals with a mutation in APOB (c.1672C>T/p.Arg558Ter). Gray denotes individuals with a mutation in PCSK9 (c.94G>A/p.Glu32Lys). Circles indicate females, whereas squares indicate males.

A family tree is illustrated in Figure 1. The proband (II-1) was born from non-consanguineous marriage. During the study period, his mother (I-2) was alive (71 years old), although his father (I-1) had died due to a car accident. Both parents (I-1 and I-2) had been diagnosed with diabetes. The patient has a wife (II-4, 47 years old) diagnosed with dyslipidemia (initial LDL cholesterol level, 223 mg/dL), the mother of whom (I-4) died from myocardial infarction. Radiographic assessment of her Achilles tendon revealed a size of 8.3 and 7.5 mm (right and left). No cutaneous xanthomas were identified. Thus, she did not satisfy the clinical criteria for FH in Japan at this moment (10). Rosuvastatin (5 mg/dL) and ezetimibe (10 mg/dL) were introduced to her, which reduced her LDL cholesterol level to 96 mg/dL. Moreover, the proband has a younger brother (II-2) and younger sister (II-3). Accordingly, his younger brother (II-2) also exhibited very low levels of LDL cholesterol with a history of Graves' disease, which is currently being treated. The proband has a daughter (III-1, 18 years old) and a son (III-2, 14 years old), both of whom had no apparent disease histories.

Clinical and genetic backgrounds are summarized in Table 1. There is no sign of lipid storage, xanthoma or other skin changes in the all of the subjects. Among the exome regions of the 21 genes known to be associated with Mendelian lipid disorders, a novel protein-truncating mutation (c.1672C>T/p.Arg558Ter) was found in *APOB* in four family members, including the proband (II-1), his younger brother (II-2), his daughter (III-1), and his son (III-2). No other mutations were found to have caused the patient's condition. Apart from this mutation, we identified a pathogenic mutation (i.e., FH) in proprotein convertase subtilisin/kexin type 9 (*PCSK9*, c.94G>A/p.Glu32Lys) in his wife (II-4) and son (III-2). This mutation has been considered one of the most common pathogenic mutations among the Japanese population (11). Thus, his son (III-2) exhibited concurrent FH and FHBL. Notably, his son (III-2) exhibited hypobetalipoproteinemia, rather than hypercholesterolemia without any elevation in transaminases. Tendon and cutaneous xanthomas we not identified in his son (III-2).

**Table 1:** Clinical and genetic characteristics of the pedigree.

**Subject (gender)**	I-2^*^(female)	**II-1 (male)**	**II-2 (male)**	**II-4 (female)**	**III-1 (female)**	**III-2 (male)**
*APOB* genotype	W/W	M1/W	M1/W	W/W	M1/W	M1/W
*PCSK9* genotype	W/W	W/W	W/W	M2/W	W/W	M2/W
Age (yr)	71	50	49	47	18	14
LDL cholesterol (mg/dL)	83	19	38	223	42	42
Triglycerides (mg/dL)	114	20	25	105	26	26
HDL cholesterol (mg/dL)	72	62	58	59	86	78
ApoA1 (mg/dL)	NA	149	NA	141	168	167
ApoB (mg/dL)	NA	22	NA	155	28	26
ApoE (mg/dL)	NA	2.9	NA	4.4	3.8	2.5
ApoE phenotype	3/2	3/2	3/3	4/3	4/3	3/3
CETP (μg/mL)	1.1	2.2	1.3	1.9	1.9	2.1
AST (IU/L)	17	38	45	17	19	18
ALT (IU/L)	13	51	76	9	14	9
γ-GTP (IU/L)	19	28	29	11	11	11
Direct bilirubin (mg/dL)	0.2	0.3	0.2	0.3	0.2	0.2
Indirect bilirubin (mg/dL)	0.3	0.3	0.3	0.3	0.3	0.2

APOB genotype: W = wild type, M1 = c.1672C>T/p.Arg558Ter.

PCSK9 genotype: W = wild type, M2 = c.94G>A/p.Glu32Lys.

ApoA1, apolipoprotein A1; ApoB, apolipoprotein B; ApoE, apolipoprotein E; CETP, cholesterylester transfer protein; AST, aspartate aminotransferase; ALT, alanine aminotransferase; γ-GTP, γ-glutamyl transpeptidase; NA, not available.

^*^Eicosapentaenoic acid (EPA) and docosahexaenoic acid (DHA) 2 g/day.

We didn't provide any treatments for the patients with FHBL1, including the proband because no drug can has been currently approved as a treatment for NAFLD in Japan. Meanwhile, we will provide continuous surveillance for liver dysfunction, including imaging for their liver.

## Discussion

We herein report a rare family with coexistence of FHBL1 caused by a novel protein truncated mutation in APOB and FH caused by a known pathogenic mutation in PCSK9. In his family, individuals with FHBL1 exhibited low levels of ApoB-containing lipoproteins, including LDL cholesterol and triglycerides, whereas another patient with FH exhibited high levels of LDL cholesterol. Interestingly, one patient with FHBL1 and FH exhibited low levels of ApoB-containing lipoproteins, suggesting that phenotypes of FH were masked by a presence of FHBL1.

It may be a matter of discussion whether the subject III-2 is FH. In this context, the guideline of FH in Japan that has just been updated stipulates that a patient with a pathogenic variant as FH can be diagnosed as FH regardless of other situations (10). So, now we can diagnose him as FH using the new guideline. This is probably due to the situation where a substantial number of patients with FH (with a pathogenic variant as FH) who do not exhibit high LDL cholesterol usually have other causes that can reduce LDL cholesterol. In that situation, if the condition lowering LDL cholesterol is removed (e.g., hyperthyroidism), then their LDL cholesterol levels is expected to increase. In this case (III-2), if a gene therapy for FHBL1 will be available in the future, then his LDL cholesterol is expected to increase to the point of FH. In addition, We expect that the LDL cholesterol level of III-2 (FHBL and FH) will increase because (1) he is now in his puberty where LDL cholesterol is generally lowered (12), and (2) he has a pathogenic variant as FH. Accordingly, we may need to treat his LDL-C in the future. However, it is possible that his LDL cholesterol level remains quite low due to his concomitant situation of FHBL1. We will monitor his LDL cholesterol as well as his liver enzyme to see if his LDL cholesterol needs to be treated and how. Ezetimibe, instead of statins may be better to be used in this case, since the production of APOB-containing lipoprotein in his liver should be already disturbed.

To date, a few cases with the same situation have been described (13). Given that a truncating mutation in *APOB* can neutralize the extreme elevation of LDL cholesterol among patients with FH, targeting this pathway could be one of the promising strategies to reduce not only LDL cholesterol but also their triglycerides and Lp(a) among patients with FH.

Although patients with FHBL1 have been shown to be lower risk for cardiovascular disease (14), at least a portion of the patients with FHBL1 have been shown to be complicated with non-alcoholic fatty liver disease (NAFLD) (15, 16). Accordingly, correct diagnosis is crucial since NAFLD could be a lethal disease. It is unclear why his son (III-2) who had FHBL and FH did not exhibit liver dysfunction, despite the same mutation with the proband (II-1); however, we speculate that a “second hit,” such as aging would be needed to exceed the threshold of this situation. In any way, appropriate surveillance for liver dysfunction should be considered among patients with FHBL1. These discussions make us wonder what is the right balance between accumulating lipids in the liver and those in the blood. In terms of anti-atherosclerotic properties, accumulating lipids in the liver, rather than in the blood appears to be quite good (14). On the other hand, accumulating lipids in the liver sometimes causes NAFLD, and it may lead to reduce their life span. However, data so far suggest that only a part of the subjects with FHBL1 exhibit NAFLD, while cardioprotective effects have been established in population-based level. Accordingly, accumulation of lipids in the liver, rather than those in the blood may be a preferable situation. In other words, FHBL1 may be a better situation compared with a condition of FH.

In conclusion, we report an extremely rare family where the phenotypes of FH were negated by the coexistence of FHBL1 caused by a truncating mutation in *APOB*.

## Patient perspective

We believe that this patient will be free from coronary artery disease due to lowered APOB-containing lipoproteins, although regular surveillance for NAFLD is required.

## Data availability statement

The data analyzed in this study is subject to the following licenses/restrictions: Due to the nature of this article, IRB of Kanazawa University did not agree for their data to be shared publicly, so supporting data is not available. Requests to access these datasets should be directed to HT, ht240z@sa3.so-net.ne.jp.

## Ethics statement

The studies involving human participants were reviewed and approved by IRB of Kanazawa University. The patients/participants provided their written informed consent to participate in this study.

## Author contributions

KS, HT, MK, and TI contributed to the patient's care and toward preparing the manuscript and approved the final version of the manuscript. All authors contributed to the article and approved the submitted version.

## Funding

We have received a grant from JSPS KAKENHI (21K08066).

## Conflict of interest

The authors declare that the research was conducted in the absence of any commercial or financial relationships that could be construed as a potential conflict of interest.

## Publisher's note

All claims expressed in this article are solely those of the authors and do not necessarily represent those of their affiliated organizations, or those of the publisher, the editors and the reviewers. Any product that may be evaluated in this article, or claim that may be made by its manufacturer, is not guaranteed or endorsed by the publisher.
